# Tight Coupling of Metabolic Oscillations and Intracellular Water Dynamics in *Saccharomyces cerevisiae*

**DOI:** 10.1371/journal.pone.0117308

**Published:** 2015-02-23

**Authors:** Henrik Seir Thoke, Asger Tobiesen, Jonathan Brewer, Per Lyngs Hansen, Roberto P. Stock, Lars F. Olsen, Luis A. Bagatolli

**Affiliations:** 1 MEMPHYS—Center for Biomembrane Physics, Department of Biochemistry and Molecular Biology, University of Southern Denmark, Campusvej 55, DK5230, Odense M, Denmark; 2 MEMPHYS—Center for Biomembrane Physics, Department of Physics, Chemistry and Pharmacy, University of Southern Denmark, Campusvej 55, DK5230, Odense M, Denmark; 3 Instituto de Biotecnología, Universidad Nacional Autónoma de México (IBt-UNAM), Av. Universidad 2001, Cuernavaca, Morelos, 62210, Mexico; 4 CelCom group, Department of Biochemistry and Molecular Biology, University of Southern Denmark, Campusvej 55, DK5230, Odense M, Denmark; Texas A&M University, UNITED STATES

## Abstract

We detected very strong coupling between the oscillating concentration of ATP and the dynamics of intracellular water during glycolysis in *Saccharomyces cerevisiae*. Our results indicate that: i) dipolar relaxation of intracellular water is heterogeneous within the cell and different from dilute conditions, ii) water dipolar relaxation oscillates with glycolysis and in phase with ATP concentration, iii) this phenomenon is scale-invariant from the subcellular to the ensemble of synchronized cells and, iv) the periodicity of both glycolytic oscillations and dipolar relaxation are equally affected by D_2_O in a dose-dependent manner. These results offer a new insight into the coupling of an emergent intensive physicochemical property of the cell, i.e. cell-wide water dipolar relaxation, and a central metabolite (ATP) produced by a robustly oscillating metabolic process.

## Introduction

The oscillatory behavior of many biological processes has been studied for decades. Examples include slow genetic oscillations of circadian rhythms [1], periodic pattern formation in embryogenesis [2], oscillating cytoskeletal structure in mechano-sensitive hair bundles in the auditory system and, at the single cell level, the oscillations of Min gene products in *Escherichia coli* that dynamically determine the site of cell division, among others [3].

The oscillatory nature of glycolysis in *Saccharomyces cerevisiae* becomes apparent when unmasked by inhibition of respiration. As cells utilize glucose supplied in the medium, glycolysis products accumulate and disappear following a well-known waveform. Oscillations can be measured in real time following the intrinsic fluorescence of reduced nicotine adenine dinucleotide, NADH [4,5]. Oscillations of other intracellular glycolytic intermediates [6], CO_2_ [7], mitochondrial potential, ATP and intracellular pH [8] have been observed, suggesting the existence of underlying coupling mechanisms. Glycolytic oscillations are a property of single cells [5] but, at high cellular density, they become macroscopic since cells are quickly and robustly synchronized via diffusing metabolites.

Attempts to understand oscillating glycolysis have taken the form of models of a few to tens of enzymatic reactions and some rate-controlling steps (e.g. phosphofructokinase). However, a kinetically equivalent proposal of control of oscillations by sugar transport has established that the origin of oscillations is diffuse and not governed by a single reaction, whether chemical transformation or sugar transport [9]. All models have in common that they are premised on mass action kinetics. In other words, they rely on the assumption that the intracellular milieu is, at a relevant scale, a homogeneous environment where diffusing chemical species, consumed and produced by enzymes at particular rates (with requisite delays, [10]), are responsible for the periodic accumulation and disappearance of measured metabolites.

This view of the intracellular environment as a nanoscale replica of dilute systems, however, is probably missing crucial information [11,12]. It does not consider the peculiar physicochemical properties of the intracellular aqueous phase, treating it as a featureless isotropic environment in which chemical species move and react. This view has an honorable history going back to van't Hoff's insight that solutes could, in dilute conditions, be treated with formalisms successfully applied to gases. A view that incorporates the altered colligative properties of the intracellular aqueous milieu would, however, provide a more comprehensive picture [11]. Considering what is known of the behavior of hydrogel materials [13], coherent macroscopic behavior in cells or sets of cells (physiology) can be conceived as the result of dynamical coupling of mechanochemical (i.e. viscoelastic) properties of the medium to chemical transformations (metabolism).

Water constitutes the most abundant component of the cell. Its unusual properties as a polar solvent have been recognized as part and parcel of life processes [14]. While van't Hoff's insight has proven invaluable, it reaches its limit of usefulness when water itself is strongly impacted by solutes (that is, deviates from tractable ideality). Furthermore, water in the cell is not just a medium where reactions occur but an active participant, e.g. many cellular reactions are condensations that produce water, or hydrolytic ones that consume it. It has been long known that the cellular environment is highly crowded with very little water exhibiting the properties of dilute solutions (for example, transverse relaxation times in muscle, [15]). Even in simple model systems, NMR studies of interfacial water indicate that its properties are quite dissimilar to dilute systems (see [16]). Many cellular processes, such as secretion and endocytosis, have been productively modeled by responsive hydrogels [17]. An explicit treatment of the dynamics of intracellular water should, therefore, provide elements for a more detailed structural, mechanistic and dynamical understanding of the coherence of cellular behavior, that is, the coupling between chemical and mechanical levels of description [18]. For such a treatment to be feasible, however, time resolved information of intracellular water behavior is essential. The aim of our investigation was to study water dynamics in real time in oscillating yeast cells using fluorescence methods.

For this, we made use of a series of polarity sensitive fluorescent molecules (**d**imethyl**a**mino**n**aphthalene, DAN probes) consisting of ACDAN, PRODAN, and LAURDAN [19]. These probes are sensitive to solvent general properties (dielectric constant, index of refraction) and, more importantly to our purpose, to solvent (water) relaxation phenomena occurring during their nanosecond fluorescence lifetime, measured through the Generalized Polarization (GP) function [19–21], see Material and Methods section below. The three probes constitute a series exhibiting different affinities for hydrophobic/hydrophilic environments [22].

Because of their partition properties and shared response to solvent dipolar relaxation, the DAN probes were our chosen sensors to monitor heterogeneities in the collective dynamics of the most abundant intracellular dipoles, namely, water molecules. We took advantage of the oscillatory behavior of glycolysis to add a temporal dimension (phase and period) that would allow us to establish correlations between the fluorescence dynamics of the probes and metabolism. We focused on the phase relationships between fluorescence oscillations of the DAN probes and those of glycolytic metabolites, and perturbed the system with heavy water to quantify the response of the oscillators to determine the extent of coupling. The scale of the observations was varied from measurement of the macroscopic oscillations of populations of cells by fluorometry, to the subcellular level using fluorescence microscopy. *The molecular level at which glycolytic oscillations are generated and controlled is not the subject of this study; our subject is the effect of these oscillations at the cellular level*.

## Materials and Methods

### Chemicals

The aptamer switch probe S10 was synthesized by VBC Biotech (Vienna). Hexokinase from yeast was purchased from USB Products (OH, USA); pyruvate kinase from rabbit muscle was from Sigma (Munich, Germany); Texas Red Dextran from Life Technologies Europe (Nærum, Denmark); 3,3’-diethyloxacarbocyanine iodide (DiOC_2_(3)) from Molecular Probes (Eugene, OR, USA). 8-anilino-1-naphthalenesulfonic acid (ANS), 6-propionyl-2-dimethylaminonaphthalene (PRODAN) and 6-lauroyl-2-dimethylaminonaphthalene (LAURDAN) were from Invitrogen (Denmark). 6-acetyl-2-dimethylaminonaphthalene (ACDAN) was a generous gift from Dr. Parkson L. G. Chong (Dept. of Biochemistry, Temple University School of Medicine, Philadelphia, Pennsylvania). All other chemicals were from Sigma (Munich, Germany).

### Yeast strains and growth

The yeast strain used in this study was the *Saccharomyces cerevisiae* diploid strain X2180 grown essentially as described in Poulsen et al [7], i.e. under semiaerobic conditions at 30°C on a rotary shaker, 180 rpm, in a medium containing 10 g/l glucose, 6.7 g/l yeast nitrogen base (Bacto) and 100 mM potassium phthalate (Aldrich, Germany) at pH 5.0. Yeast cells were harvested at the point when glucose was depleted as measured with a glucose test strip (Macherey-Nagel, Düren, Germany). The cells were washed twice with 100 mM-potassium phosphate buffer (Merck, Germany), pH 6.8 (centrifugation, 5 min at 5000 rpm, GSA, Sorvall), resuspended in the same buffer to a cell density of 10% by weight and starved for 3 h on a rotary shaker at 30°C.

### Measurements of intracellular ATP concentration

The intracellular ATP concentration was measured using an aptamer-based ATP nanosensor as described previously [23,24]. Briefly, a calibration curve was constructed for the ATP sensor in an aqueous solution containing a mixture of ATP and ADP where the total concentration of ATP plus ADP is 4 mM to match the total concentration of ATP in a yeast cell [6]. The ATP sensors were inserted into yeast cells by electroporation and the fluorescence from the sensors was recorded. The corresponding fluorescence from cells containing nanoparticles without the sensors was also recorded. These nanoparticles were also inserted by electroporation. Finally, the fluorescence from cells containing empty nanoparticles was subtracted from the fluorescence from the cells containing nanosensors and the resulting signal was transformed into ATP concentration using the calibration curve constructed for the sensor in aqueous solution.

### Labeling of yeast cells with the probes ACDAN, PRODAN and LAURDAN

2 ml of yeast cells at a density 10% by weight suspended in 100 mM potassium phosphate buffer, pH 6.8, were incubated with 5 μM of the probes at room temperature for 60–120 min (the longer times were used for incubation with LAURDAN). The cells were washed twice with potassium buffer by centrifugation at 10,000 g for 1 min in a small bench centrifuge to sediment the cells and finally suspended in fresh 100 mM potassium phosphate buffer, pH 6.8. For the D_2_O experiments the cells were, after the second wash, suspended in 100 mM potassium phosphate buffer, pH 6.8 containing 10% (v/v) or 50% (v/v) D_2_O. The cell suspensions were transferred to quartz cuvettes for immediate fluorescence measurements.

### Fluorescence cuvette measurements

Experiments were performed in an Edinburgh FL 920 spectrofluorometer using a 2 ml sample cell with constant stirring and temperature control (Quantum Northwest, WA, USA). The temperature around the sample cell was maintained at 25.00 ± 0.01°C. The samples contained unlabeled yeast cells or cells labeled with ACDAN, PRODAN, LAURDAN or DiOC_2_(3). The fluorescence signal due to NADH was measured at 450 nm (10 nm slit) with excitation at 366 nm (3 nm slit). Fluorescence emission spectra for ACDAN, LAURDAN and PRODAN were done with excitation at 366 nm (3 nm slit) and emission measured from 400 nm to 600 nm. Temporal measurements of the fluorescence of the three DAN probes (ACDAN, LAURDAN and PRODAN) and ANS in yeast cells were done with excitation at 390 nm (3 nm slit) to avoid simultaneous excitation of NADH, and with emissions at 440 nm, 450 nm or 490 nm (3 nm slit). Measurements of DiOC_2_(3) fluorescence were done with an excitation at 480 nm (3 nm slit) and emission at 600 nm (3 nm slit) using 5 μM probe as described in reference [25]. Yeast cells at a density of 10% by weight were suspended in 100 mM potassium phosphate buffer, pH 6.8.

Simultaneous measurements of DiOC_2_(3) and ACDAN fluorescence were made in a QE65000 spectrometer (Ocean Optics, Dunedin, FL) also using a 2 ml sample cell with constant stirring and temperature control (Quantum Northwest, WA, USA). Temperature was 25 ± 0.01°C. Light was supplied by two Hg-lamps (model St75; Heraeus, Hanau, Germany) powered by two NT HgSt power supplies (Duratec Analysentechnik, GmBH, Hockenheim, Germany). Light from the lamps was guided to the measuring cell using optical fibers (Rapp Optoelectronic, GmBH, Hamburg, Germany) mounted perpendicular to the emission light beam. DiOC_2_(3) was excited using a 500 nm (24 nm bandwidth) interference filter (Edmund Optics Inc., Barrington, NJ), and emission was measured as the average intensity in the wavelength range 580–620 nm. ACDAN was excited using a 355 nm (bandwidth 20 nm) interference filter (Edmund Optics Inc., Barrington, NJ) and emission was measured as the average intensity in the wavelength range 445–470 nm. All measurements were done in triplicate. Power spectra of the oscillating time series measured by fluorometry were computed using Berkeley-Madonna software (Berkeley, CA). Instrumental error (as SEM) for all fluorescence intensity measurements is on the size scale of the symbols used in the graphs.

### Fluorescence microscopy measurements

**Fig 1:**
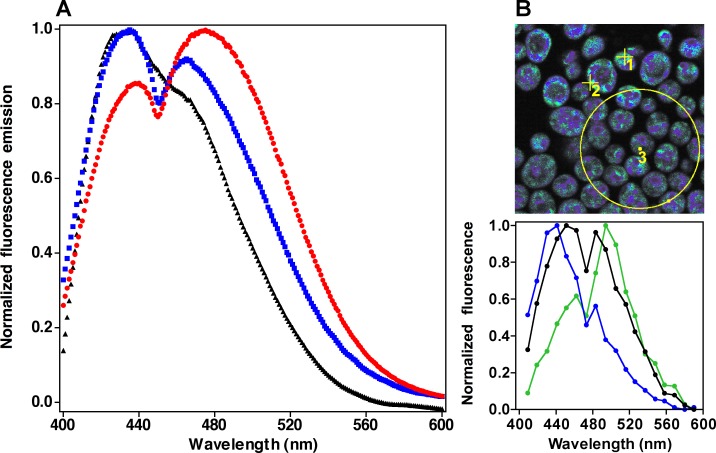
Fluorescence response of DAN probes in resting cells. Panel **A**) Emission spectra of cells labeled with ACDAN (red), PRODAN (blue) and LAURDAN (black) measured in the fluorometer. Panel **B**) Spectral image of cells labeled with ACDAN (top) with spectra (bottom) of selected regions of interest: single B region (blue, ROI 1), single G region (green, ROI 2), and the overall spectrum (black, large circle defines ROI 3). Spectral resolution in the microscope is lower than in the spectrofluorometer. Image size is 15 x 15 μm. The spectral images of PRODAN and LAURDAN are not shown.

An inverted multiphoton excitation fluorescence microscope (Zeiss LSM 510 META NLO, Carl Zeiss, Jena, Germany) equipped with a Ti:Sa MaiTai XF-W2S laser (Broadband Mai Tai with 10 W Millennia pump laser, tunable excitation range 710–980 nm, Spectra Physics, Mountain View, CA) was used in the experiments presented in Fig. 1B. The excitation wavelength for ACDAN, PRODAN and LAURDAN was 780 nm. The fluorescence signal was collected using the microscope’s meta detector in spectral mode acquisition with a resolution of 10 nm. The objective used was a 63X water immersion, NA 1.2. Acquisition of the two photon excitation fluorescence images was performed adding 300 μl of PRODAN-, ACDAN- or LAURDAN-labeled resting yeast cells at a density of 10% by weight (suspended in 100 mM potassium phosphate buffer, pH 6.8) to an 8-well plastic chamber (Lab-tek Brand Products, Naperville, IL). The chamber containing the sample was placed in the microscope and the images were acquired a few minutes later to allow the cells to sink to the bottom of the well.

Time resolved NADH, ACDAN and PRODAN fluorescence intensity images were obtained in a custom built multiphoton excitation fluorescence microscope described elsewhere [26]. The objective used was a 40X water immersion objective NA 1.29 and the excitation laser source was a Ti:Sa laser (HPe MaiTai DeepSee, Spectra Physics, Mountain View, CA). NADH excitation was at 740 nm and the DAN probes were excited at 810 nm.

Emission light was split to two detectors (H7422 PMT, Hammamatsu, Denmark) by a 460 nm long pass dichroic mirror and then filtered by 520 ± 17.5 nm and 438 ± 12 nm band pass filters (both from AHF Analysen Technik AG, Tübingen, Germany). The images were anayzed using custom MATLAB (Mathworks, Inc., Natik, MA, USA) scripts and ImageJ (NIH, Bethesda, MD, USA). Power spectra were computed by first applying a moving average over 5 images, smoothing the data and then subtracting the average of all the images. These experiments were performed by adding 200 μl of oscillating yeast (from a sample whose oscillations where simultaneously measured in a fluorometer, as indicated above) into an 8-well plastic chamber (Lab-Tek, Naperville IL) and taking images in intervals of 3 seconds in a time window of 5–15 minutes after addition. Power spectra of the oscillating time series measured by fluorescence microscope were computed with a custom MATLAB program using the built-in FFT (Fast Fourier Transform) algorithm. The GP oscillations were computed using a customized MATLAB script. We first calculated the mean intensity of each image (or ROI) in the green and blue channels. Based on these data sets the script calculates the GP and then applies the same smoothing methods described above, i.e. first a moving average of 5 steps followed by subtraction of the average. All measurements were done in triplicate.

### Sensitivity of DAN probes to water relaxation

As discussed elsewhere in detail [20,21,27], the dynamics of water dipoles in the vicinity of the DAN moiety of the fluorophores cause fast fluorescence responses due to their reorientation in the presence of the transient excited state dipole. The energy for reorientation is drawn from the excited state of the fluorophore, shifting its emission spectrum to longer wavelengths and reducing its quantum yield (i.e., emission intensity). The DAN probes have no ionizable groups and it has been shown that they are not responsive to changes in the activity of protons in the range of pH 4 to 10 [27,28].

The DAN probes used in this study constitute a series in which the fluorescent group is coupled to progressively longer hydrophobic chains, thus exhibiting different affinities for hydrophobic/hydrophilic environments [22]. The shortest of these (C_2_, ACDAN), is essentially insensitive to membrane-associated water dynamics since it partitions to hydrophilic environments. The next (C_3_, PRODAN), partitions to both membranes and surrounding water. The most hydrophobic probe contains a lauroyl chain (C_12_, LAURDAN), which places it squarely within bilayers as its solubility in water is negligible. These partition properties allow the exploration of a broad range of cellular environments, which is important to establish whether we are measuring a global cellular phenomenon.

In the experiments reported in this paper we focused on the two main fluorescent parameters of the DAN probes: i) emission intensity at a given wavelength (which depends on the quantum yield of the probes), and ii) the Generalized Polarization function [21,27,28], which requires simultaneous measurement of two wavelengths.

The generalized polarization (GP) function was originally introduced as an analytical method to quantitatively determine the relative amounts and temporal fluctuations of two distinct lipid phases when they coexist in a model membrane, for reviews see [21,27]. This function was originally defined as:

$${\text{GP = I}}_{\text{B}}{\text{− I}}_{\text{R}}{\text{/ I}}_{\text{B}}{\text{+ I}}_{\text{R}}$$ (1)

where I_B_ and I_R_ are the measured fluorescence intensities under conditions in which a wavelength (or a band of wavelengths) B (for blue shifted) and R (for red shifted) are both observed using a given excitation wavelength. Being a weighted difference, the values of the GP must fall within −1 and 1; the lower the value the greater the extent of relaxation (or bathochromic shift of the spectrum). This definition is formally identical to the classical definition of fluorescence polarization, in which B and R represent two orthogonal orientations of the observation polarizers in the fluorometer. The advantage of the GP function for the analysis of the spectral properties of the DAN probes is derived from the well-known properties of the classical polarization function, which contains information on the interconversion between two different “states” of the emitting dipole of the fluorophore. In the original studies, the LAURDAN GP was shown to distinguish between the extent of water relaxation in solid-ordered (*s*_*o*_) and liquid-disordered (*l*_*d*_) phases in phospholipid membranes [21,27,28]; importantly, it was possible to distinguish fluctuations in the GP values *only* when the two lipid phases coexist.

In the GP function as used here, the two states correspond to the unrelaxed and relaxed environments sensed by the probes. Our approach to the study of intracellular water dynamics in yeast therefore constitutes a generalization of the use of the GP function. In this case, however, we explore fluctuations in water relaxation throughout the cell rather than in just membrane-associated water. The approach is justified by the observation that the more water-soluble DAN probes clearly detect two main fluorescence populations which correspond to unrelaxed and relaxed states of the probe, as shown in Fig. 1A. Therefore, oscillations of the GP function in the cell yielding the measured changes in the intensity of emission (quantum yield) of the probes at any given wavelength can be explained *only if solvent relaxation is the dominant mechanism*.

### Polyethylene glycol (PEG), crowding and DAN fluorescence experiments

PEG 8000 was mixed with MilliQ water to the desired concentrations. An aliquot of probe (ACDAN, PRODAN) in ethanol was then added to reach a final probe concentration of 1 μM, ensuring that the ratio of probe to PEG monomers was small, in the order 10^−6^–10^−7^. All samples were rotated overnight to ensure full mixing. Fluorescence emission of each solution was measured in an ISS Chronos fluorometer (Champaign, IL, USA) at 20°C with excitation at 374 nm using a diode as excitation source. The slits were 1 nm. The emission maxima were determined by fitting a Gaussian curve to each emission spectrum in the vicinity of its maximum and the relative quantum yields were calculated by dividing the integrated fluorescence intensity in the range of 400–600 nm. All measurements were done in triplicate.

## Results

### Fluorescence response of the DAN probes in cells

Yeast cells labeled with DAN probes exhibit emission spectra with two discernible populations with maxima in the green (G, ≈490 nm) and blue (B, ≈440 nm; Fig. 1A). This indicates the presence of two populations of fluorophores exposed to different extents of water dipolar relaxation, i.e. unrelaxed (B) and relaxed (G). The fluorescence intensity ratio between these two bands (G/B) decreases in the order ACDAN>PRODAN>LAURDAN, correlating with the decreasing affinity of the probes for hydrophilic environments (ACDAN is the most water soluble). The response of the probes in yeast differs from the single fluorescence emission maximum in pure water centered at ≈520 nm (S1 Fig.).

The response of the DAN probes observed in the fluorometer could be the product of two distinct populations of cells differentially relaxing the probes to the two different maxima or, alternatively, of most cells containing two regions of distinct dipolar relaxation. Two photon excitation fluorescence microscopy experiments support the latter alternative (Fig. 1B). Regions of different maxima in single cells are apparent as the scale is progressively varied from the subcellular to single cells to groups of cells. ACDAN and PRODAN label the cells throughout and show distinct blue (region 1) emission areas surrounded by more diffuse bathochromically shifted (green, region 2) emission areas. When the overall spectrum of several cells (region 3) is measured, it adds up to the spectrum obtained in the fluorometer. The spatial distribution of LAURDAN is not far from that observed with the other two probes, although its overall fluorescence emission is shifted to shorter wavelengths (S2 Fig.).

The responses of ACDAN and PRODAN, the more water soluble probes, were studied in a model crowded system consisting of aqueous solutions of the hydrophilic polymer polyethylene glycol (PEG 8000, S3 Fig.). The emission maximum (λ_max_) significantly shifts quasi-linearly towards shorter wavelengths as PEG concentration increases, and relative quantum yield (QY) concomitantly increases. There are no differences between PRODAN and ACDAN in their responses in polymer solutions. The form of this response cannot be solely accounted for by using an equilibrium model of probe binding with two states (bound/unbound), but must include global coupling to water dynamics in the medium (manuscript in preparation). This indicates that the DAN probes respond to the degree of solvent relaxation, which diminishes as crowding increases.

### Glycolytic oscillations

**Fig 2:**
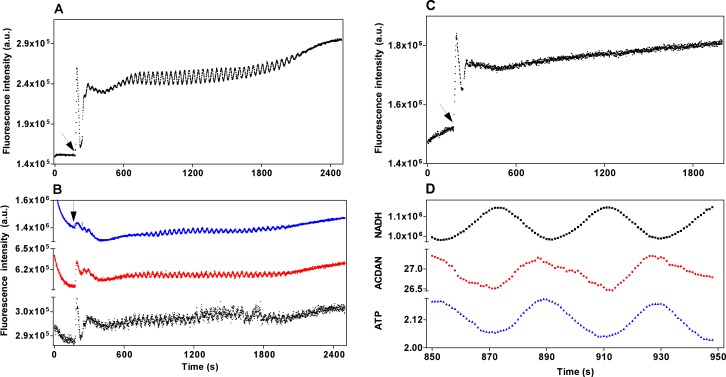
Oscillatory behavior of glycolysis and DAN probes in the fluorometer. Panel **A**) Oscillations of NADH. Panel **B**) Oscillations of ACDAN (red), PRODAN (blue) and LAURDAN (black). Panel **C**) Non-oscillatory behavior of ANS labeled cells. Panel **D**) Phase relationships: ACDAN and NADH are expressed as fluorescence intensity, ATP is plotted in concentration units (mM). The arrows in panels **A**), **B**), and **C**) indicate the time of addition of 30 mM glucose followed by 5 mM KCN.

Oscillations in glycolysis were induced by addition of glucose and potassium cyanide (KCN) to glucose-depleted dense cell suspensions. Typical NADH oscillations are shown in Fig. 2A and oscillations with an indistinguishable period in yeast labeled with all three DAN probes in Fig. 2B. The largest oscillation amplitude was observed with PRODAN while LAURDAN produced the smallest. Cells labeled with the protein/membrane-binding probe anilinonaphthalene-8-sulfonate (ANS) did not oscillate (Fig. 2C, see Discussion). Glycolytic oscillations can also be generated using Argon to maintain the yeast suspension under anaerobic conditions and partially remove acetaldehyde [7]. A comparable result to the oscillations shown in Fig. 2A and 2B was obtained when the addition of CN^−^ was replaced by bubbling Argon through the sample (results not shown).

We then investigated the phase relationship between the macroscopic (cuvette) oscillations of the water soluble DAN probes and the glycolytic metabolites NADH and ATP. It is not possible to measure the oscillations of all three variables simultaneously due to, for example, the overlapping emission spectra of NADH and the DAN probes. Therefore, phase relationships were inferred indirectly from the following measurements: First we simultaneously measured the fluorescence of NADH and the carbocyanine dye DiOC_2_(3) (data not shown) and confirmed that they oscillate in phase as previously reported in [8]. Next, we measured simultaneously the fluorescences of DiOC_2_(3) and the DAN probe and showed that they oscillate exactly 180° out of phase (S4 Fig.). Finally, we measured the phase relationship between NADH and ATP oscillations and showed that these also oscillate 180° out of phase (indicated in Fig. 2D), in accord with previous reports [6,24]. From these measurements we can establish phase relationships between NADH, DAN probe fluorescence and ATP as shown in Fig. 2D. Note here that the DAN probe fluorescence oscillates in phase with ATP.

**Fig 3:**
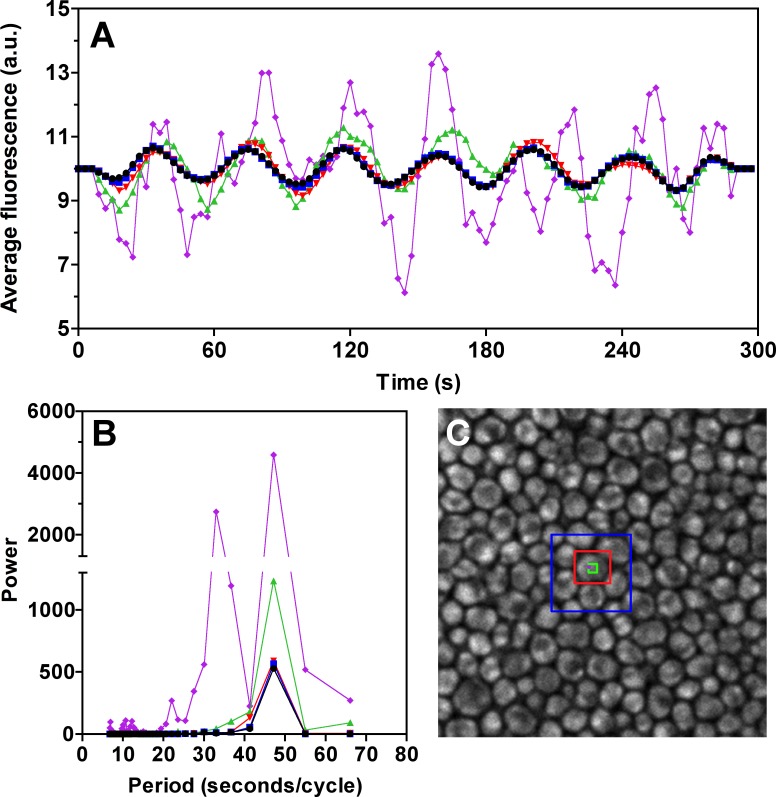
NADH oscillations in the microscope. Panel **A**) Running average of NADH oscillations at different scales. Black, whole image; Blue, 7 cells; Red, 1 cell; Green, 7x7 intracellular pixels; Purple, single pixel. Panel **B**) Power analysis of oscillations in each region. Panel **C**) Picture of NADH fluorescence (438±12 nm) with color-coded regions of interest. Pixel size is 0.1 μm, image corresponds to a field of 25.6 x 25.6 μm.

**Fig 4:**
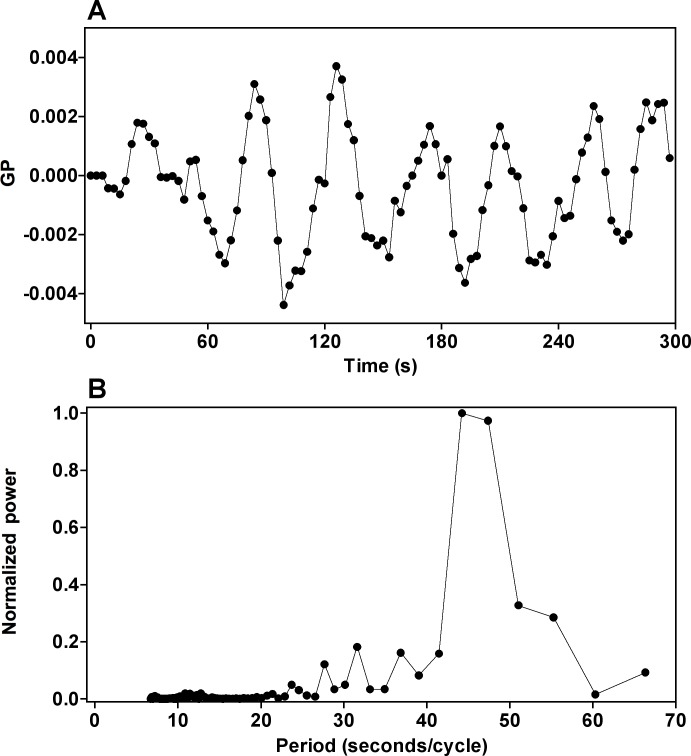
Oscillations in the Generalized Polarization (GP) function of ACDAN in oscillating cells. **Panel A**) Oscillations of the values correspond to the weighted difference of the intensity of emission at the maxima shown in Fig. 1. Panel **B**) Power spectrum of the frequency of the GP oscillations. The GP was calculated as described in supplementary text (equation 1). Note that the GP function indicates that the blue (440 nm) and green (490 nm) emission intensities oscillate in a correlated manner, as would be expected if both the B and G regions of Fig. 1 were oscillating synchronously. The same behavior was observed with PRODAN.

Oscillations of NADH concentration span all measured size scales, as established by multiphoton excitation microscopy in progressively smaller regions of interest (ROIs), ranging from many cells to a single intracellular pixel (Fig. 3). The fluorescence intensity (S5 Fig.) and GP function (Fig. 4, obtained by simultaneous measurements at 440 and 490 nm) of the DAN probes also oscillate synchronously with ATP. When glycolysis is blocked with iodoacetate (S6 Fig.), oscillations of the active metabolites (ATP, NADH) and the DAN probes disappear in a correlated manner. Furthermore, we observed a diminishing quantum yield of PRODAN, indicating an increase in overall dipolar relaxation.

### The effect of heavy water (D_2_O) on the oscillations of NADH, ATP and the DAN probes

**Fig 5:**
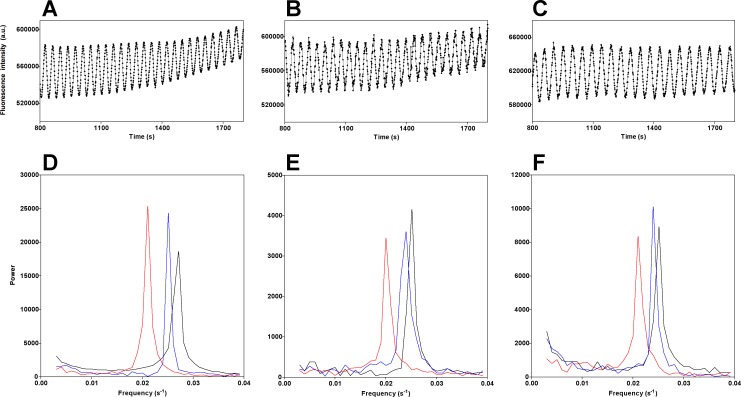
The effect of D_2_O on NADH, ACDAN and PRODAN oscillations. The top panels show NADH oscillations in the presence of no D_2_O (A), 10% D_2_O (B) and 50% D_2_O (C). The bottom panels show the power spectra of the oscillations of NADH (D), ACDAN (E) and PRODAN (F) with increasing concentrations of D_2_O (black 0%, blue 10% and red 50%).

We suspended the yeast cells in increasing concentrations of D_2_O (0%, 10% and 50% v/v) and measured the oscillations of NADH, ACDAN and PRODAN. There is a small but significant decrease in NADH oscillation frequency in the presence of 10% D_2_O and a large decrease in frequency (≈ 20%) in the presence of 50% D_2_O (Fig. 5A, B and C), apparent in the power spectrum representation (Fig. 5D). We observed exactly the same responses in the oscillatory behavior of ACDAN (Fig. 5E) and PRODAN (Fig. 5F) in these conditions. D_2_O had the same effect when cells were labeled with DiOC_2_(3) (S7 Fig.).

## Discussion

### Water dipolar relaxation, D_2_O, crowding and the DAN probes

Pure water relaxes at an extremely fast rate [29], in picoseconds, yielding the observed ACDAN peak at 520 nm. Compared to water, D_2_O is denser, has higher freezing and boiling temperatures, and is more viscous. However, ACDAN and PRODAN in pure H_2_O and D_2_O display exactly the same emission peak (S1 Fig.). In other words, *in bulk* (i.e. when dipoles reorient much faster than the excited state lifetime) the DAN probes are "blind" to any differences between them: both solvents draw the maximum possible energy from the excited state of the probes. The DAN probes become sensitive to differences in water dipolar relaxation in situations where it is hindered by, for example, hydrophillic polymers such as PEG, where ACDAN experiences a progressive blue shift (lower relaxation) and an increase in quantum yield as crowding increases (S3 Fig.). That the DAN probes in yeast discriminate two intracellular populations with emission maxima at ≈440 nm (B) and ≈490 nm (G) suggests that the state of water in the cells is differentially restricted in its ability to reorient in response to the excited state dipole of the fluorophores. In resting cells, 50% D_2_O does not alter the emission spectrum (the B and G regions) of any of the DAN probes (S8 Fig.), consequently, its effect is on the temporality of the oscillations, not on the probes.

In interfacial environments water motion (relaxation) is slowed down. During the nanosecond lifetime of the DAN probes, differences in the dynamics *of the processes that involve solvent relaxation* become apparent and are "sensed" by them. A well-studied example is the effect of D_2_O on a membrane phase transition studied with the LAURDAN GP function; slower relaxation dynamics are observed at and above the solid-ordered ➔ liquid-disordered transition [21,27]. This cannot be the consequence of a chemical reaction because the transition involves no compositional changes. The simplest explanation is that the greater mass of the ensemble of (membrane-associated) D_2_O molecules reorienting (sensed by LAURDAN) as the supramolecular structure changes is enough to slow down the transition.

### Oscillation dynamics (period and phase) and the effect of D_2_O

Oscillations in fluorescence intensity and GP of the DAN probes and ATP concentration are rigorously in phase. The non-oscillatory behavior of ANS -a probe of proteins and membranes more sensitive to local polarity of specific binding sites rather than overall water dipolar relaxation dynamics (ANS is soluble but virtually non-fluorescent in water; [30])- argues against oscillations originating in interactions not mediated by water. Iodoacetate (a blocker of glycolysis) causes a correlated disappearance of all oscillations (of NADH, ATP and DAN probes) and, as ATP is depleted, a bathochromic spectral response of the probes. These observations indicate that water properties are coupled with the metabolic process that generates ATP. It is important to note that ATP/ADP by themselves in a crowded solution do not affect ACDAN fluorescence (S9 Fig.), suggesting that it is the cellular environment that is responsible for the oscillatory behavior of the probe.

Heavy water has many biological effects [31]. Its impact on cytoskeletal dynamics is well documented although quantitative explanations are still under investigation [32]. The lower frequency of glycolytic oscillations in the presence of D_2_O might be conceived as a consequence of the fact that deuterated compounds are chemically more stable and that the rates of reactions involving deuterated bonds can be slower. However, while this would be an apparently straightforward explanation for the *chemical transformations* very much in the spirit of van’t Hoff, it fails to explain why the oscillations of the DAN probes occur at all, and why they remain synchronous with the slowed down ATP oscillations. It is important to stress that it is the *frequency* of all oscillations that is affected by D_2_O, not the response of the probes (S8 Fig.).

We propose that a more comprehensive explanation requires consideration of the well-known fact that the presence of deuterium affects the rates of reactions even if deuterated bonds are not themselves involved (*cf*. membrane phase transitions mentioned above). This effect is termed the secondary isotope effect [31]; with deuterated water in the medium, it seems reasonable to conclude that the entire nanoenvironment where oscillating glycolysis occurs involves some degree of structure that is *dynamically* affected by the addition of a small amount of extra mass per molecule in the most abundant class of molecules. Again, the lack of oscillatory behavior in cells labeled with ANS argues against oscillations originating in protein interactions with the DAN probes.

All wavelengths of emission of the DAN probes (the B and G regions of the cells) are oscillating. This was confirmed by fluorescence microscopy measurement of the fluorescence intensity changes of the DAN probes (S5 Fig.) and their GP function (Fig. 4), which concordantly show that -within the resolution of our system- glycolysis and dipolar relaxation oscillate with the same frequency at all observed scales. Furthermore, oscillations of the GP function yield the measured changes in the intensity (quantum yield) of emission of the probes at any given wavelength *only if solvent relaxation is the dominant mechanism*. The scale invariance and spectrum-wide nature of the oscillations suggest that the phenomenon spans the entire cell cytosol (it is intensive) and is not localized to substructures dynamically isolated from each other. Oscillations are even detected by LAURDAN, albeit with lower amplitude, in membrane-associated water.

## Conclusions

Our current framework of understanding of cellular processes relies on the premise that the cell cytosol is, at the relevant scale, like the dilute aqueous solutions in which we study biochemical processes *in vitro*. If this were true, partially water soluble probes like ACDAN and PRODAN, sensitive to dipolar relaxation dynamics, would not be expected to sense significant changes in the intracellular medium at this scale. In our view, the properties of the oscillations of the DAN probes are more consistent with the intracellular environment behaving as a responsive hydrogel, a view with very strong experimental support [13]. The study of hydrogels has traditionally relied on classical physicochemical measurements of equilibrium properties of the medium affected by crowding such as vapor pressure, swelling and shrinking. Our results provide robust direct spatial and temporal evidence of the intracellular aqueous phase as a medium exhibiting fast and coherent coupling of an intensive (scale invariant) cellular property (i.e. intracellular water relaxation) with a central metabolic process. This conclusion challenges the use of the concept of ‘normal’ diffusion, a corollary of van’t Hoff’s theory of ideal solutions, to explain and build models of integrated cellular behavior. The coupling, at multiple scales, of water dynamics to ATP levels during glycolytic oscillations may offer a more complete perspective of the category of phenomena generally described as anomalous diffusion. In fact, the very term ‘anomalous diffusion’ is an acknowledgement that the environment inhabited by intracellular molecules of interest -in this case a key product of glycolysis whose concentration oscillates- cannot be accurately described in terms of concepts derived from the chemistry of dilute solutions, or diffusion in terms of a straightforward application of the Stokes-Einstein relationship.

Considering that polymerization/depolymerization of cytoskeletal structures is strongly dependent on ATP and ATPase activity [32–34], it is reasonable to suppose that ATP acts on the overall state of the cytoskeleton and that this impacts dipolar relaxation of the aqueous phase, possibly due to changes in viscoelastic properties. As metabolism oscillates so do interfacial water dynamics; as D_2_O makes the system "heavier", all oscillations are synchronously slowed down. The chemistry and physics of the system are thus bidirectionally coupled. Solvent (water) motion has been shown to govern an important part of the energy landscape occupied by proteins, affecting catalysis [35,36] and folding [37]. The observations reported here provide a robust biological system for theoretical development and experimental testing of Erwin Schrodinger’s insight that life depends on the maintenance of a low entropy state [38,39]. The cytosol as a hydrogel, with most of its water dynamically coupled to central metabolic processes may provide the substrate where an entropic level of understanding of key processes of life can be found [40]. Probing water dynamics in real time as reported here is a valuable tool to explore the spatiotemporal integration of the physical and the biochemical in living cells. Our results anticipate future work on solvent effects on the fluorescence of DAN probes *in vivo* in response to physiological oscillatory phenomena on longer time scales (metabolic, biosynthetic, and transcriptional) [41].

## Supporting Information

S1 Fig Fluorescence of ACDAN in non-crowded environments. Raw fluorescence emission spectra of 5 μM ACDAN in pure H_2_O (black) and pure D_2_O (red). The lower peak at shorter wavelengths corresponds to the Raman effect of the solvent. The same behavior was observed for PRODAN.
(TIF)

S2 Fig Distribution of ACDAN, PRODAN and LAURDAN in resting yeast. Spectral fluorescence images of A) ACDAN, B) PRODAN and C) LAURDAN in yeast cells. The color scale corresponds to the wavelength range used in Fig. 1B. Although the spatial distributions of the three probes are fairly similar, their fluorescence responses match those observed in the fluorescence cuvette experiments shown in Fig. 1A (see text). The average size of the individual cells is approximately 3.5 μm.
(TIF)

S3 Fig Effect of crowding by polyethylene glycol (PEG 8000) on the fluorescence properties of ACDAN. The curves show the relationship between concentration of polymer (as % by weight), the wavelength of the emission maximum of the probe (λ_max_ in nm, squares) and its relative quantum yield (QY, circles). The same behavior was observed with PRODAN.
(TIF)

S4 Fig Simultaneous measurement of DiOC_2_(3) (black) and ACDAN (red) oscillations. Panel **A**) Oscillations measured in the fluorometer. Panel **B**) Phase representation of DiOC_2_(3) and ACDAN oscillations; the result of the correlation analyses (Pearson, Spearman) is at the bottom left of the graph. The same results were obtained with PRODAN.
(TIF)

S5 Fig Scale invariance of the period of ACDAN oscillations. The measurements shown are for the 520±17.5 nm channel, although oscillations were also seen in the 438±12 nm channel. Panel **A**) Fluorescence image of oscillating ACDAN labeled cells with measured regions of interest. Panel **B**) Power analysis of the running average of ACDAN oscillations within each ROI: Black, whole image (15.4 x 15.4 μm); blue (about 9 cells); red, single cell. Pixel size is 0.06 μm. The same results were seen with PRODAN.
(TIF)

S6 Fig Effect of iodoacetate on oscillatory dynamics of glycolysis and water dipolar relaxation. Panel **A**) PRODAN emission spectra of untreated resting cells (black), cells treated with KCN and glucose (blue) and with KCN, glucose and the glycolysis inhibitor iodoacetate (red). Panel **B**) Time course of fluorescence intensity of PRODAN, NADH and ATP (in mM) oscillations after exposure to iodoacetate. Glucose (30 mM) and KCN (5 mM) were added to the cell suspensions at 180 s and 240 s, respectively; once the cells were oscillating iodoacetate (20 mM) was added at 1000 s. The same phenomenon was observed for ACDAN. The data was obtained from 3 independent runs (i.e. measurements were not simultaneous).
(TIF)

S7 Fig Oscillations of DiOC_2_(3) in presence of D_2_O. Effect of D_2_O (% v/v) on oscillations in cells labeled with the carbocyanine dye DiOC_2_(3) before and after addition of 30 mM glucose (indicated by the arrow) and 5 mM KCN (60 s later) to yeast cells in the presence of 0% (black trace), 10% (blue trace) and 50% (red trace) D_2_O. Panel **A**) Time course of oscillations in the fluorometer. Panel **B**) Power spectra of the frequency of oscillations with increasing D_2_O.
(TIF)

S8 Fig Effect of D_2_O on the emission spectra of the DAN probes in resting yeast. Effect of 50% (v/v) D_2_O on the spectral response of ACDAN (top), PRODAN (middle) and LAURDAN (bottom) in resting cells as they equilibrate with D_2_O. Black, spectrum at 0 s, blue at 1250 s and red at 2500 s. Note the absence of significant spectral responses.
(TIF)

S9 Fig Measurement of ATP concentration and ACDAN fluorescence in a model crowded system comprising 25% (w/w) PEG 8000. The solution contained 10 mM phosphate, 25 mM Na_2_SO_4_, 5 mM MgCl_2_, 6 mM ADP, 6 mM phosphoenolpyruvate, 10 mM glucose and 100 nM ATP switch probe (bottom trace, black) or 5 μM ACDAN (top trace, red) at pH 6.8. At the first arrow 10 units of pyruvate kinase were added to the solution and at the second arrow 10 units of hexokinase were added. Note the increase in the ATP sensor signal as ATP is generated by ADP phosphorylation, and the decrease to baseline as it is consumed by hexokinase. Unlike in oscillating yeast cells, throughout the cycle of ATP production and consumption the ACDAN signal remains unaltered. The ATP switch probe was excited at 580 nm (3 nm slit) and emission measured at 610 nm (3 nm slit). ACDAN was excited at 366 nm and emission measured at 490 nm. Temperature was 25 ± 0.01°C.
(TIF)
