# Supplementary figures and images for: Tight Coupling of Metabolic Oscillations and Intracellular Water Dynamics in Saccharomyces cerevisiae
Source: PLoS One. 2015 Feb 23;10(2):e0117308. doi: 10.1371/journal.pone.0117308 (PMC4338026; doi:10.1371/journal.pone.0117308)

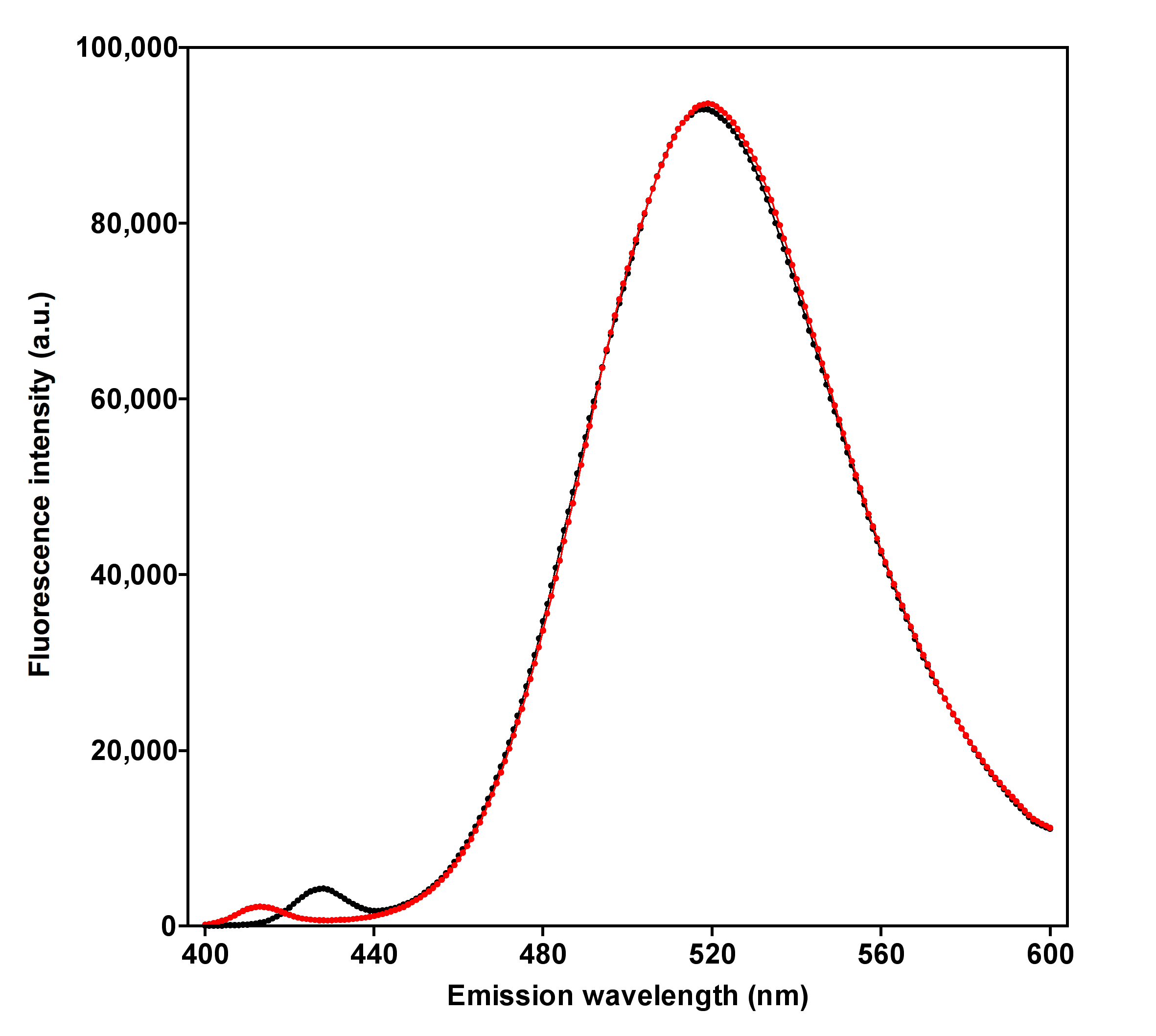

Supplement: S1 Fig — Raw fluorescence emission spectra of 5 μM ACDAN in pure H2O (black) and pure D2O (red). The lower peak at shorter wavelengths corresponds to the Raman effect of the solvent. The same behavior was observed for PRODAN. (TIF) [file pone.0117308.s001.tif]

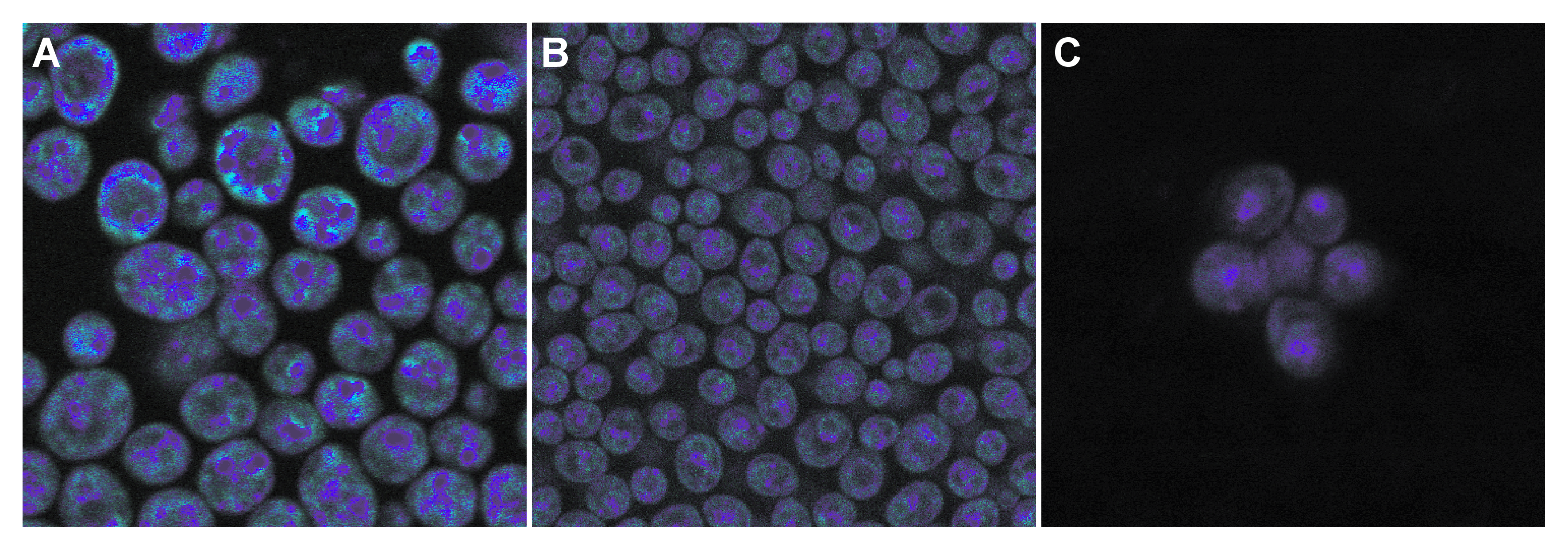

Supplement: S2 Fig — Spectral fluorescence images of A) ACDAN, B) PRODAN and C) LAURDAN in yeast cells. The color scale corresponds to the wavelength range used in Fig. 1B. Although the spatial distributions of the three probes are fairly similar, their fluorescence responses match those observed in the fluorescence cuvette experiments shown in Fig. 1A (see text). The average size of the individual cells is approximately 3.5 μm. (TIF) [file pone.0117308.s002.tif]

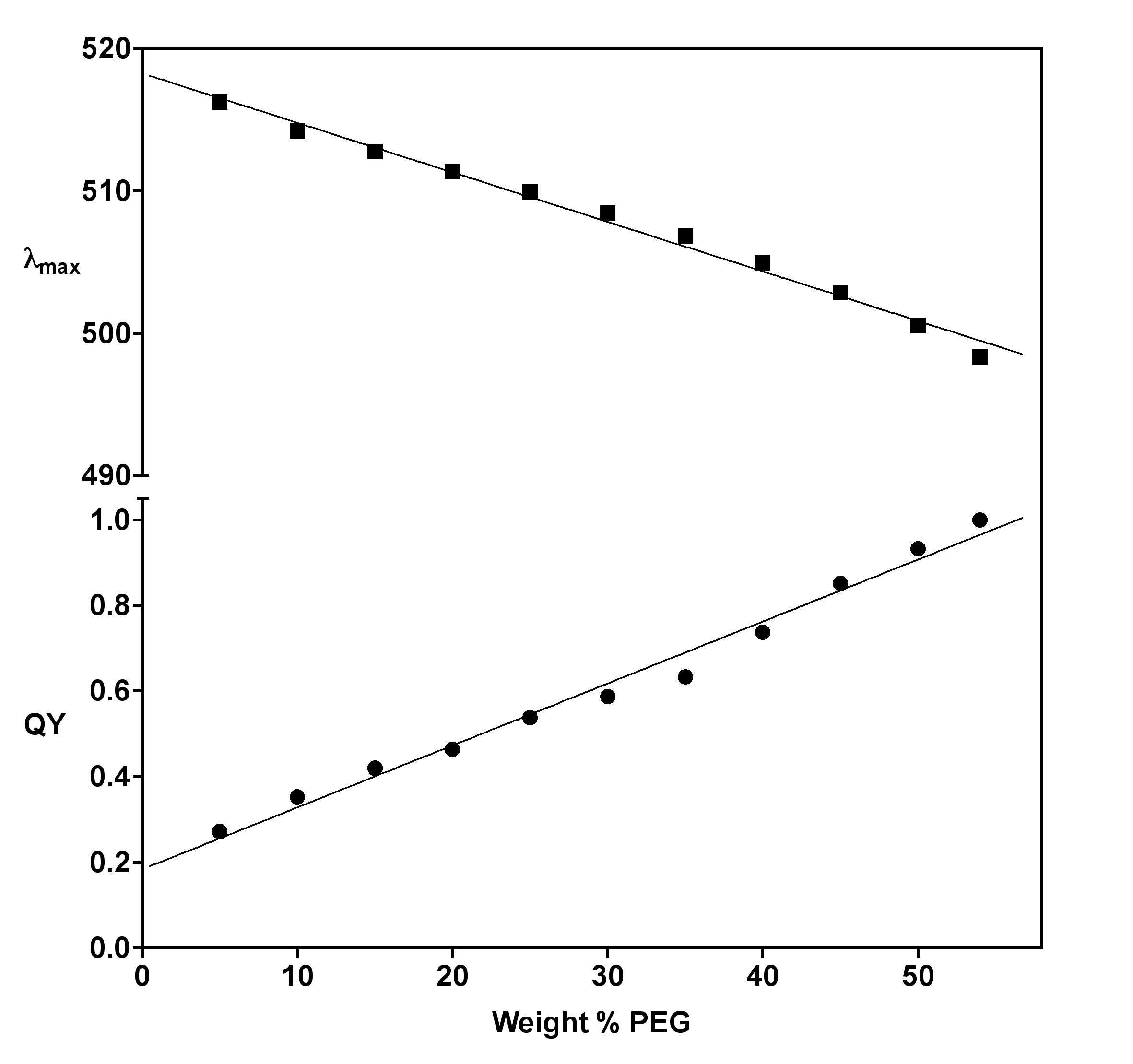

Supplement: S3 Fig — The curves show the relationship between concentration of polymer (as % by weight), the wavelength of the emission maximum of the probe (λmax in nm, squares) and its relative quantum yield (QY, circles). The same behavior was observed with PRODAN. (TIF) [file pone.0117308.s003.tif]

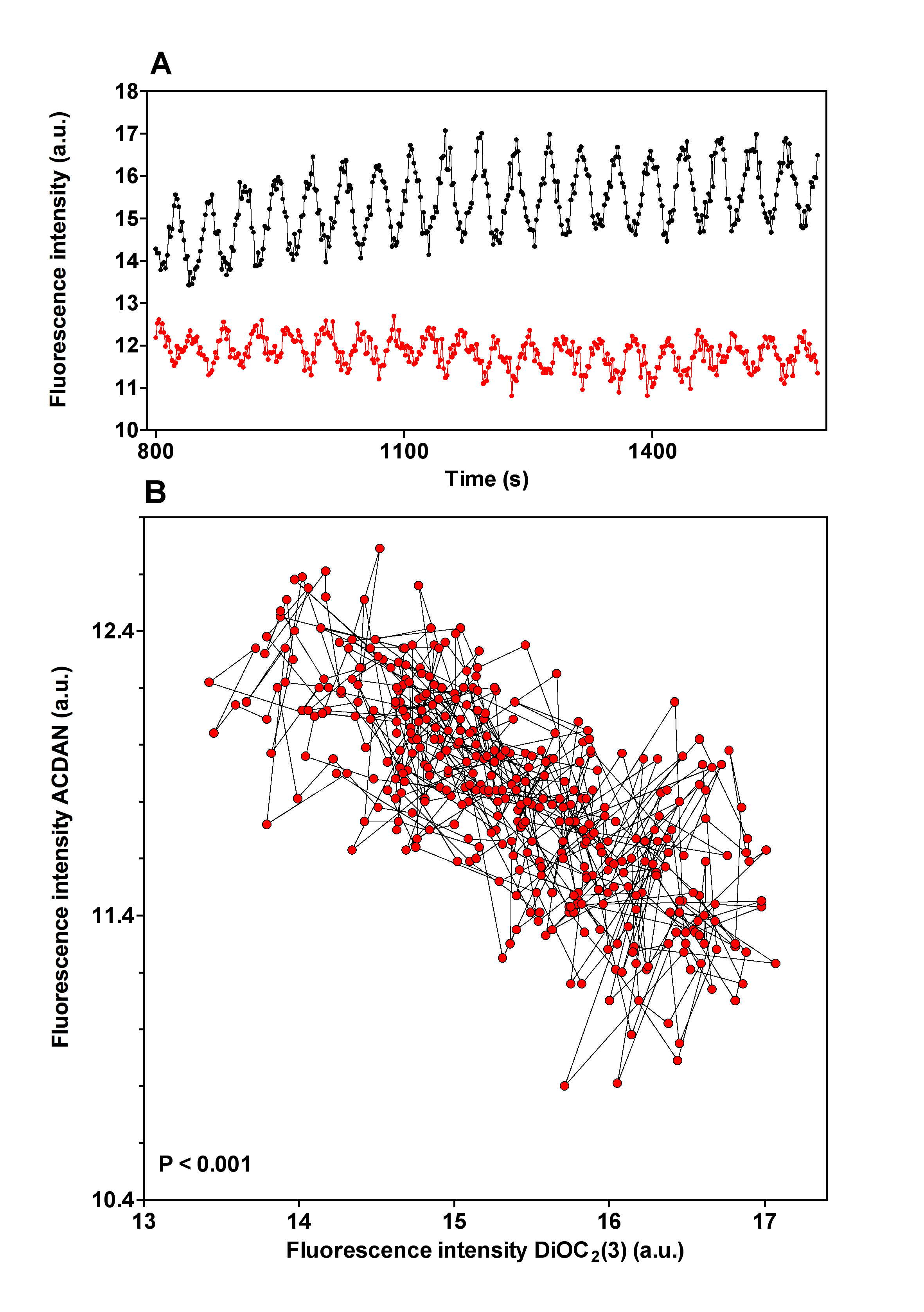

Supplement: S4 Fig — Panel A) Oscillations measured in the fluorometer. Panel B) Phase representation of DiOC2(3) and ACDAN oscillations; the result of the correlation analyses (Pearson, Spearman) is at the bottom left of the graph. The same results were obtained with PRODAN. (TIF) [file pone.0117308.s004.tif]

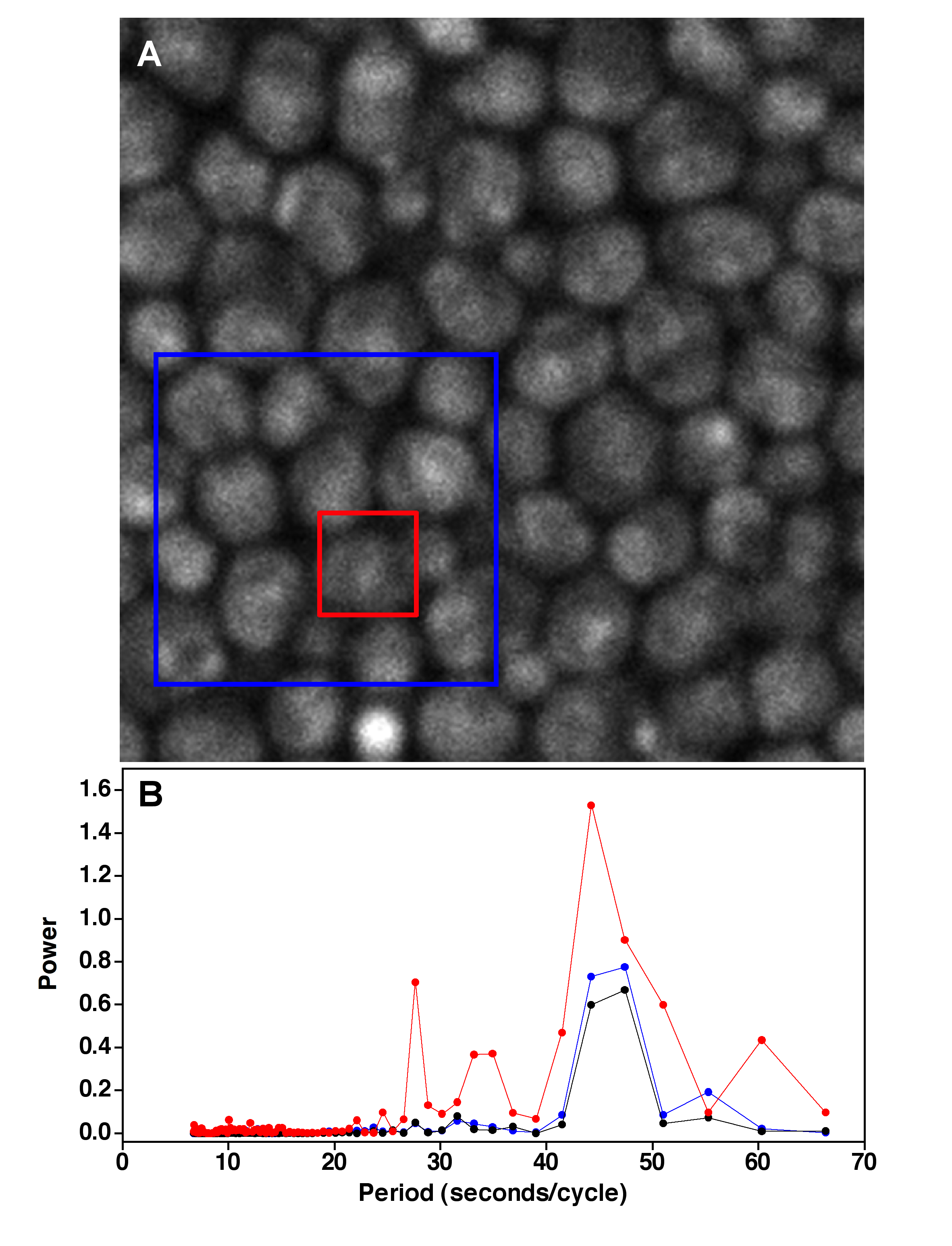

Supplement: S5 Fig — The measurements shown are for the 520±17.5 nm channel, although oscillations were also seen in the 438±12 nm channel. Panel A) Fluorescence image of oscillating ACDAN labeled cells with measured regions of interest. Panel B) Power analysis of the running average of ACDAN oscillations within each ROI: Black, whole image (15.4 x 15.4 μm); blue (about 9 cells); red, single cell. Pixel size is 0.06 μm. The same results were seen with PRODAN. (TIF) [file pone.0117308.s005.tif]

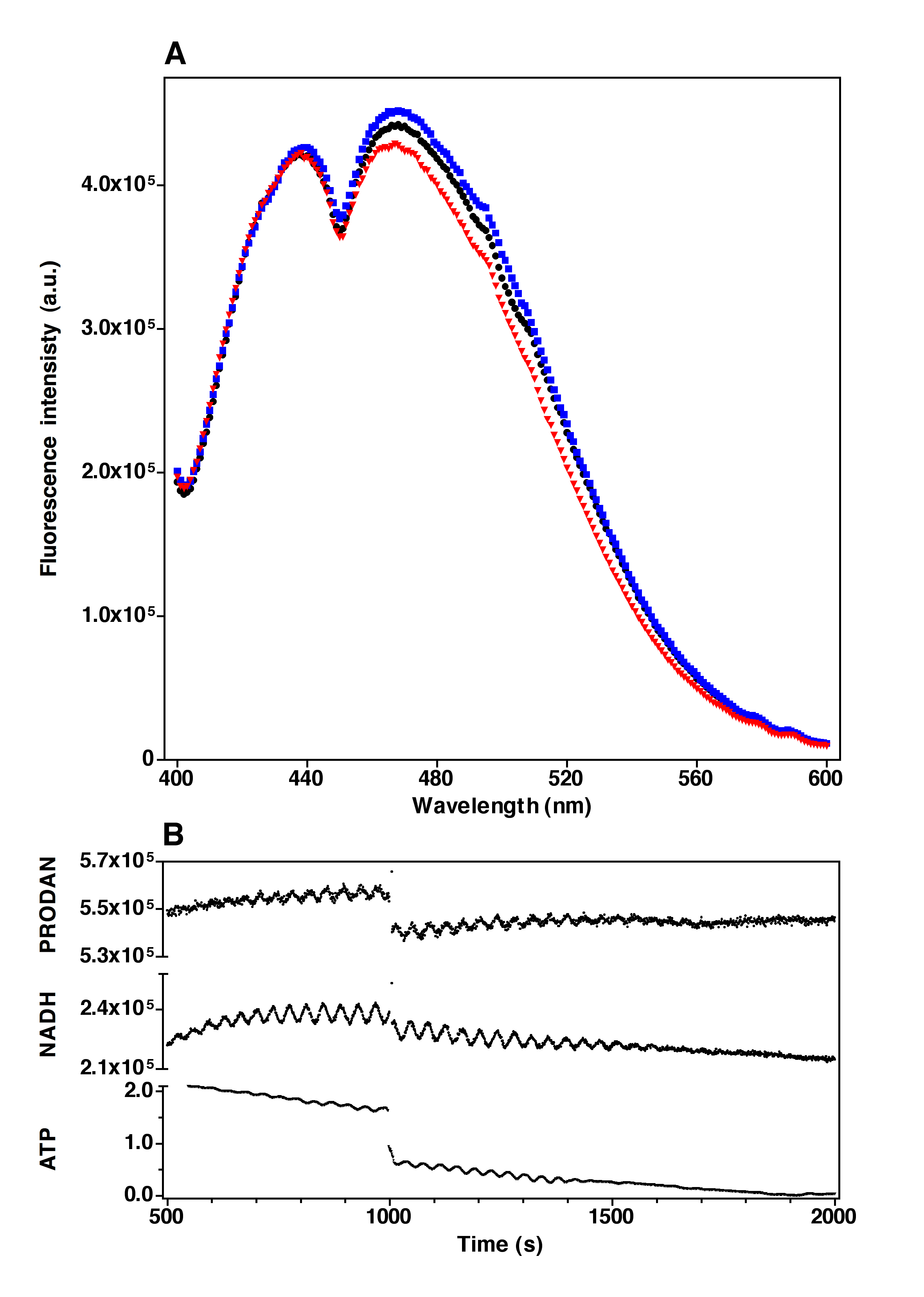

Supplement: S6 Fig — Panel A) PRODAN emission spectra of untreated resting cells (black), cells treated with KCN and glucose (blue) and with KCN, glucose and the glycolysis inhibitor iodoacetate (red). Panel B) Time course of fluorescence intensity of PRODAN, NADH and ATP (in mM) oscillations after exposure to iodoacetate. Glucose (30 mM) and KCN (5 mM) were added to the cell suspensions at 180 s and 240 s, respectively; once the cells were oscillating iodoacetate (20 mM) was added at 1000 s. The same phenomenon was observed for ACDAN. The data was obtained from 3 independent runs (i.e. measurements were not simultaneous). (TIF) [file pone.0117308.s006.tif]

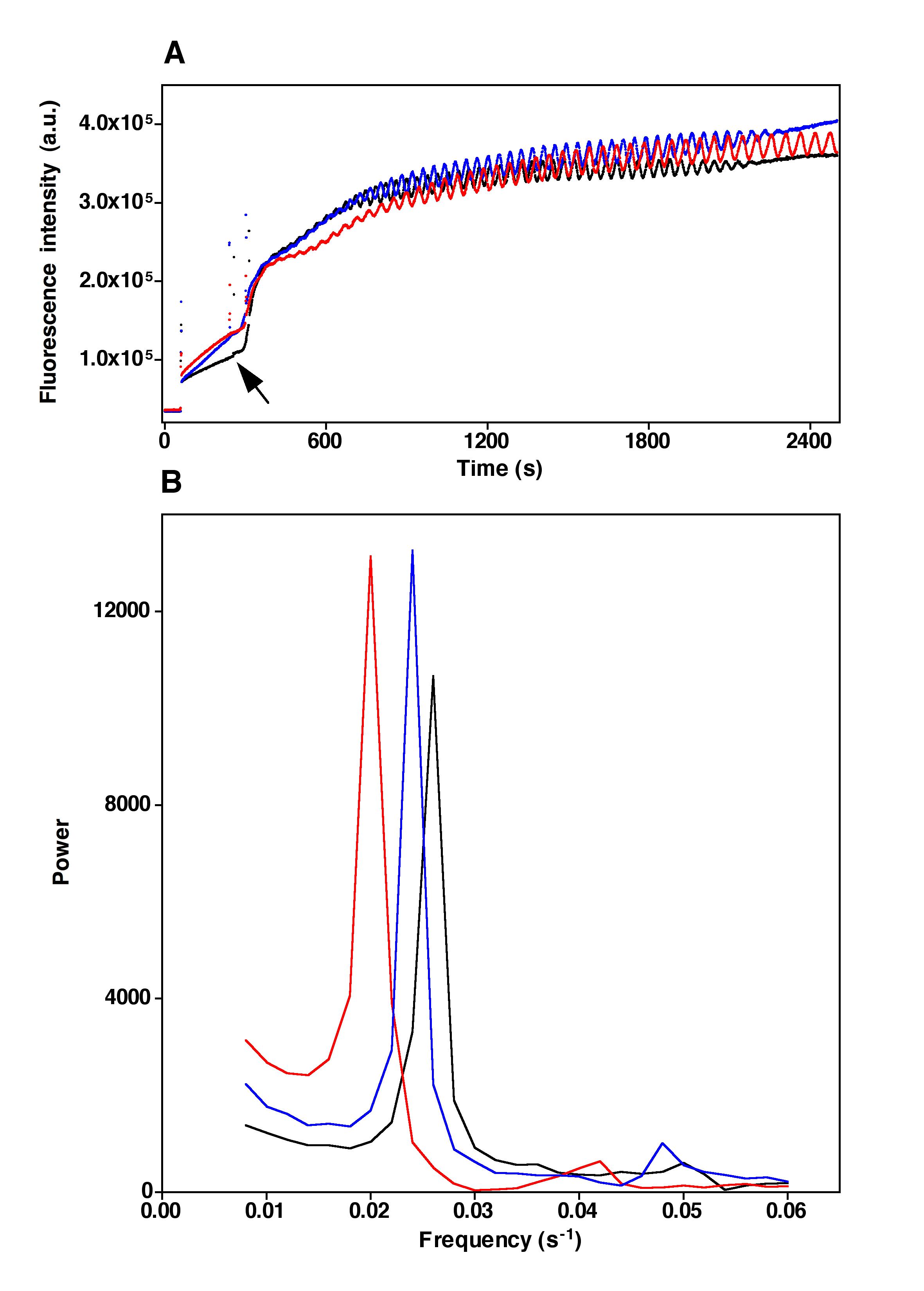

Supplement: S7 Fig — Effect of D2O (% v/v) on oscillations in cells labeled with the carbocyanine dye DiOC2(3) before and after addition of 30 mM glucose (indicated by the arrow) and 5 mM KCN (60 s later) to yeast cells in the presence of 0% (black trace), 10% (blue trace) and 50% (red trace) D2O. Panel A) Time course of oscillations in the fluorometer. Panel B) Power spectra of the frequency of oscillations with increasing D2O. (TIF) [file pone.0117308.s007.tif]

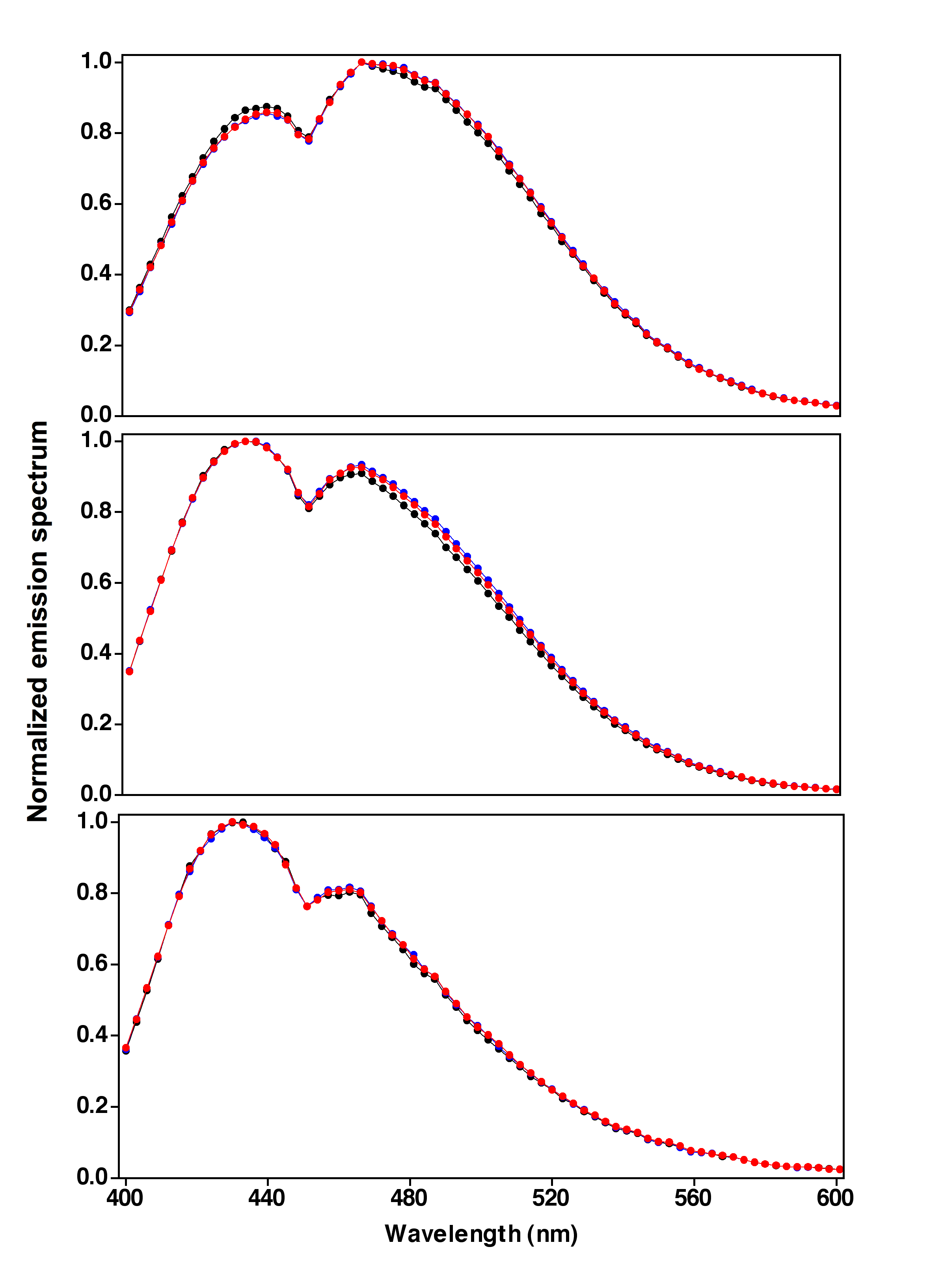

Supplement: S8 Fig — Effect of 50% (v/v) D2O on the spectral response of ACDAN (top), PRODAN (middle) and LAURDAN (bottom) in resting cells as they equilibrate with D2O. Black, spectrum at 0 s, blue at 1250 s and red at 2500 s. Note the absence of significant spectral responses. (TIF) [file pone.0117308.s008.tif]

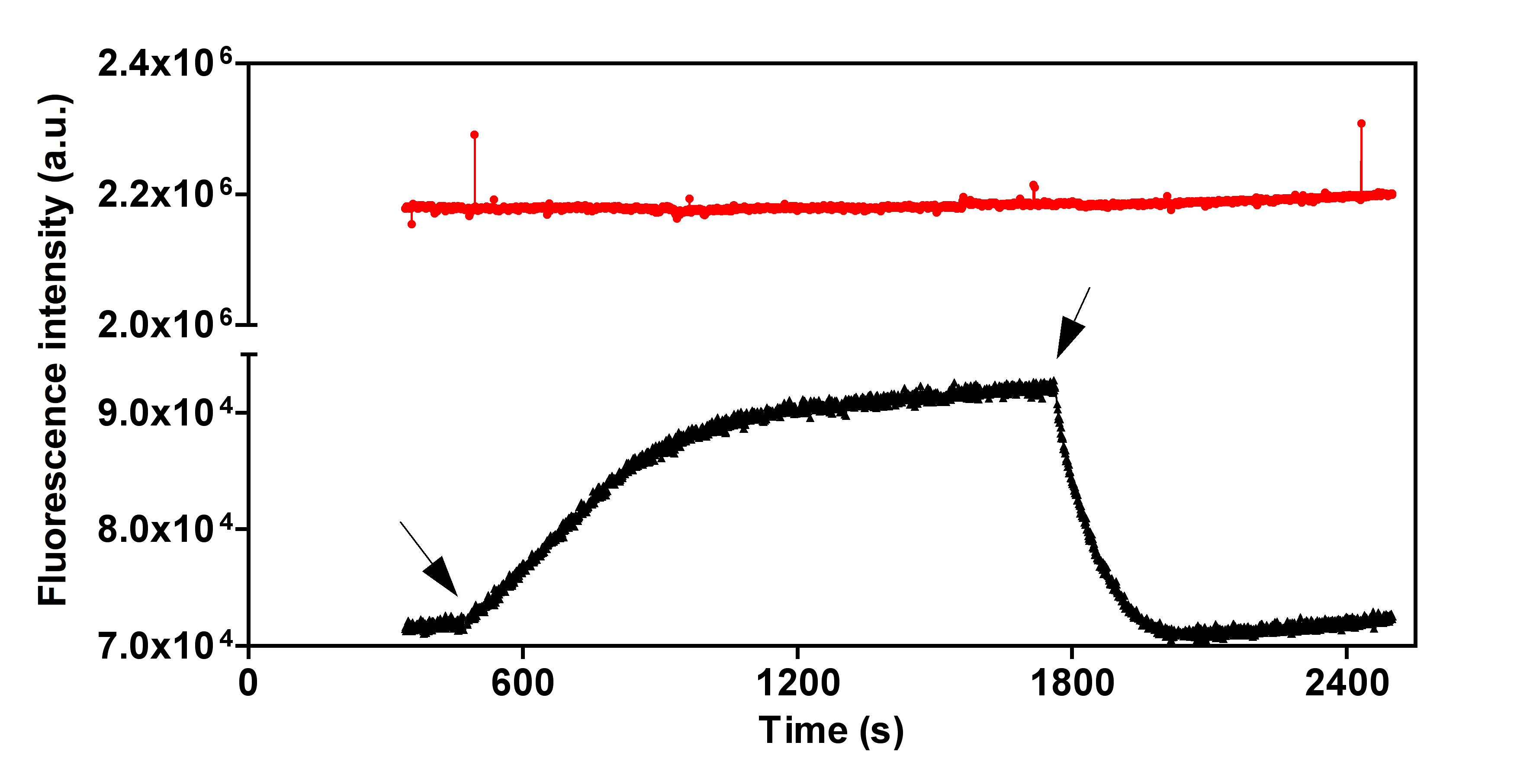

Supplement: S9 Fig — The solution contained 10 mM phosphate, 25 mM Na2SO4, 5 mM MgCl2, 6 mM ADP, 6 mM phosphoenolpyruvate, 10 mM glucose and 100 nM ATP switch probe (bottom trace, black) or 5 μM ACDAN (top trace, red) at pH 6.8. At the first arrow 10 units of pyruvate kinase were added to the solution and at the second arrow 10 units of hexokinase were added. Note the increase in the ATP sensor signal as ATP is generated by ADP phosphorylation, and the decrease to baseline as it is consumed by hexokinase. Unlike in oscillating yeast cells, throughout the cycle of ATP production and consumption the ACDAN signal remains unaltered. The ATP switch probe was excited at 580 nm (3 nm slit) and emission measured at 610 nm (3 nm slit). ACDAN was excited at 366 nm and emission measured at 490 nm. Temperature was 25 ± 0.01°C. (TIF) [file pone.0117308.s009.tif]
